# Supplementary material for: A RHNO1-ATR/Chk1 positive feedback loop sustains the DNA replication stress response
Source: bioRxiv. 2026 May 24:2026.05.22.727300. Preprint. [Version 1] doi: 10.64898/2026.05.22.727300 (PMC13228601; doi:10.64898/2026.05.22.727300)
Supplement: Supplementary file 1 [file NIHPP2026.05.22.727300V1-supplement-1.pdf]

bioRxiv preprint doi: https://doi.org/10.64898/2026.05.22.727300; this version posted May 24, 2026. The copyright holder for this preprint (which was not certified by peer review) is the author/funder, who has granted bioRxiv a license to display the preprint in perpetuity. It is made available under a CC-BY 4.0 International license.

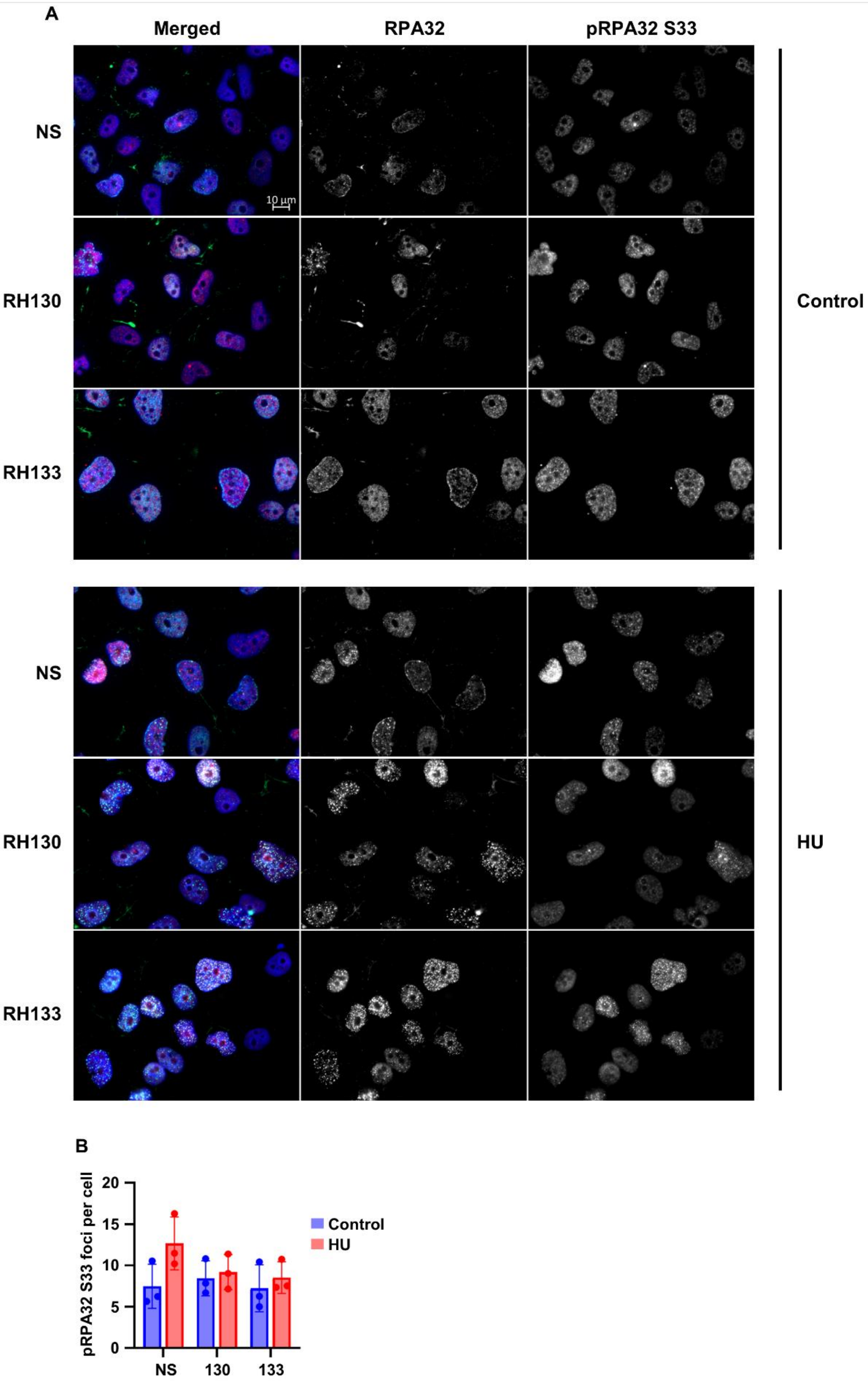

673

bioRxiv preprint doi: https://doi.org/10.64898/2026.05.22.727300; this version posted May 24, 2026. The copyright holder for this preprint (which was not certified by peer review) is the author/funder, who has granted bioRxiv a license to display the preprint in perpetuity. It is made available under a CC-BY 4.0 International license.

Supplementary figure 1 RPA32 and pRPA32 S33 foci in RHNO1-depleted OVCAR8 cell lines. **A** Representative immunofluorescence images of DAPI-stained nuclei showing RPA32 and pRPA32 S33 foci in OVCAR8 NS, RH130, and RH133 cells under control and 2.5 mM HU treatment for 5 h followed by 24 h recovery. **B** Quantification of pRPA32 S33 foci per cell. Data is presented as mean ± SD of at least three independent experiments. The scale bar represents 10 μm.

bioRxiv preprint doi: https://doi.org/10.64898/2026.05.22.727300; this version posted May 24, 2026. The copyright holder for this preprint (which was not certified by peer review) is the author/funder, who has granted bioRxiv a license to display the preprint in perpetuity. It is made available under a CC-BY 4.0 International license.

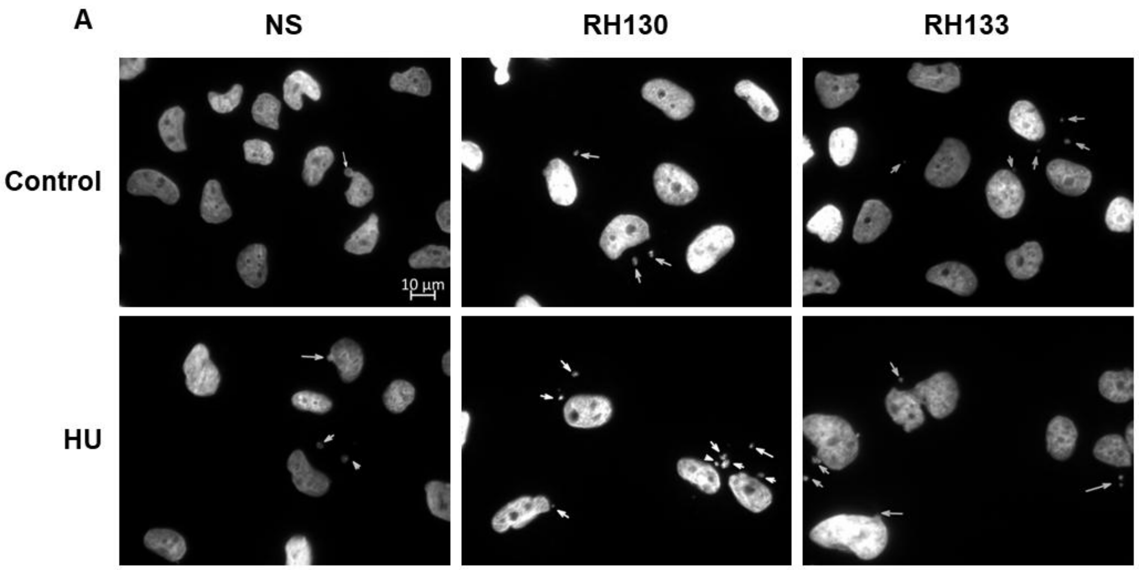

Supplementary figure 2 Micronuclei formation in RHNO1-depleted OVCAR8 cell lines. **A** Representative immunofluorescence images of DAPI-stained nuclei showing micronuclei (indicated by arrows) in OVCAR8 NS, RH130, and RH133 cells under control and 2.5 mM HU treatment for 5 h followed by 48 h recovery. The scale bar represents 10 µm.

bioRxiv preprint doi: https://doi.org/10.64898/2026.05.22.727300; this version posted May 24, 2026. The copyright holder for this preprint (which was not certified by peer review) is the author/funder, who has granted bioRxiv a license to display the preprint in perpetuity. It is made available under a CC-BY 4.0 International license.

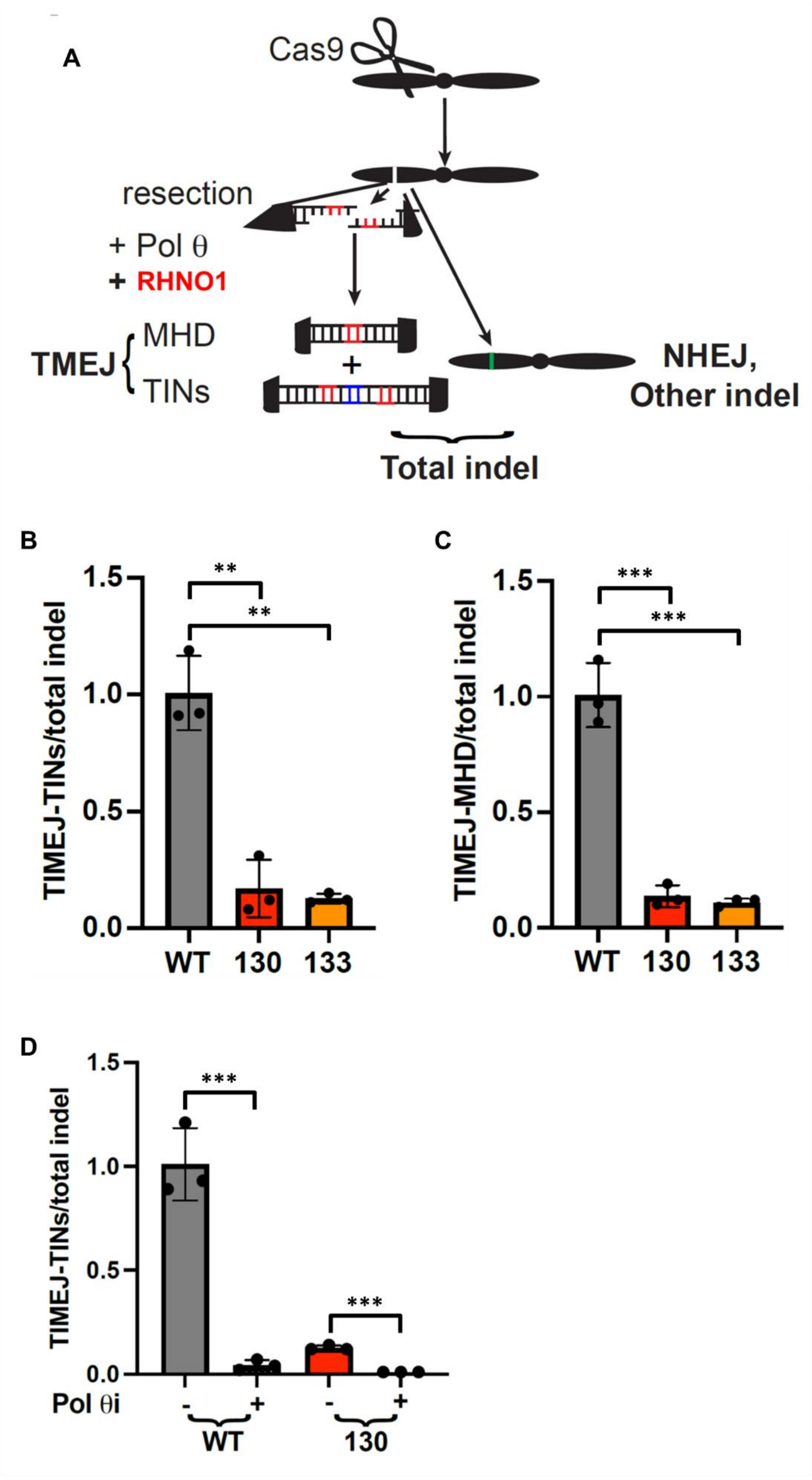

688

bioRxiv preprint doi: https://doi.org/10.64898/2026.05.22.727300; this version posted May 24, 2026. The copyright holder for this preprint (which was not certified by peer review) is the author/funder, who has granted bioRxiv a license to display the preprint in perpetuity. It is made available under a CC-BY 4.0 International license.

Supplementary figure 3 TMEJ activity in RHNO1-depleted OVCAR8 cell lines. **A** Schematic of chromosomal repair assay. Microhomology deletion (MHD) and templated insertions (TINs), as well as total insertions and deletions (indel) were measured by qPCR of DNA recovered 48 hours later after DNA double strand break induction using Cas9 at LBR gene locus. **B** and **C** The frequencies of TMEJ pathway product MHD (**B**) and TINs (**C**) normalized by total indel in OVCAR8 cell lines. **D** Quantification of TINs product in presence of DNA polymerase θ specific inhibitor, ART558. Data is presented as mean ± SD of three independent experiments.

bioRxiv preprint doi: https://doi.org/10.64898/2026.05.22.727300; this version posted May 24, 2026. The copyright holder for this preprint (which was not certified by peer review) is the author/funder, who has granted bioRxiv a license to display the preprint in perpetuity. It is made available under a CC-BY 4.0 International license.

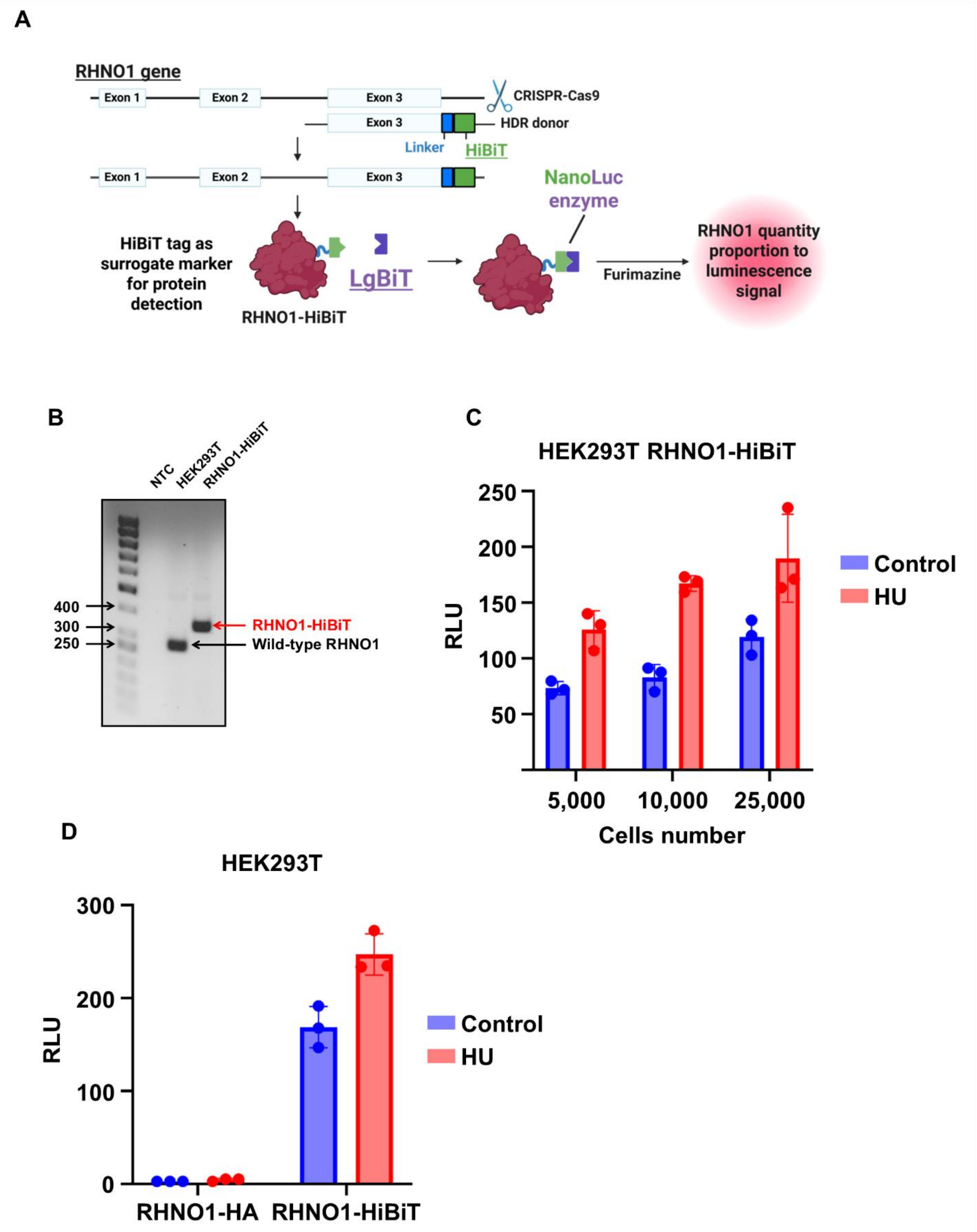

698

699

bioRxiv preprint doi: https://doi.org/10.64898/2026.05.22.727300; this version posted May 24, 2026. The copyright holder for this preprint (which was not certified by peer review) is the author/funder, who has granted bioRxiv a license to display the preprint in perpetuity. It is made available under a CC-BY 4.0 International license.

Supplementary figure 4 Generation and validation of HEK293T RHNO1-HiBiT cell line. **A** Schematic of CRISPR-Cas9 knock-in strategy for HiBiT-tagged endogenous C-terminal RHNO1. **B** Genomic PCR for the presence of HiBiT tag in RHNO1 gene. **C** HiBiT luminescence signal in HEK293T RHNO1-HiBiT in different concentration of cells. **D** Comparison of HiBiT luminescence signal between RHNO1-HA and RHNO1-HiBiT tagged cells under replication stress conditions. Data from a single experiment with three technical replicates.

bioRxiv preprint doi: https://doi.org/10.64898/2026.05.22.727300; this version posted May 24, 2026. The copyright holder for this preprint (which was not certified by peer review) is the author/funder, who has granted bioRxiv a license to display the preprint in perpetuity. It is made available under a CC-BY 4.0 International license.

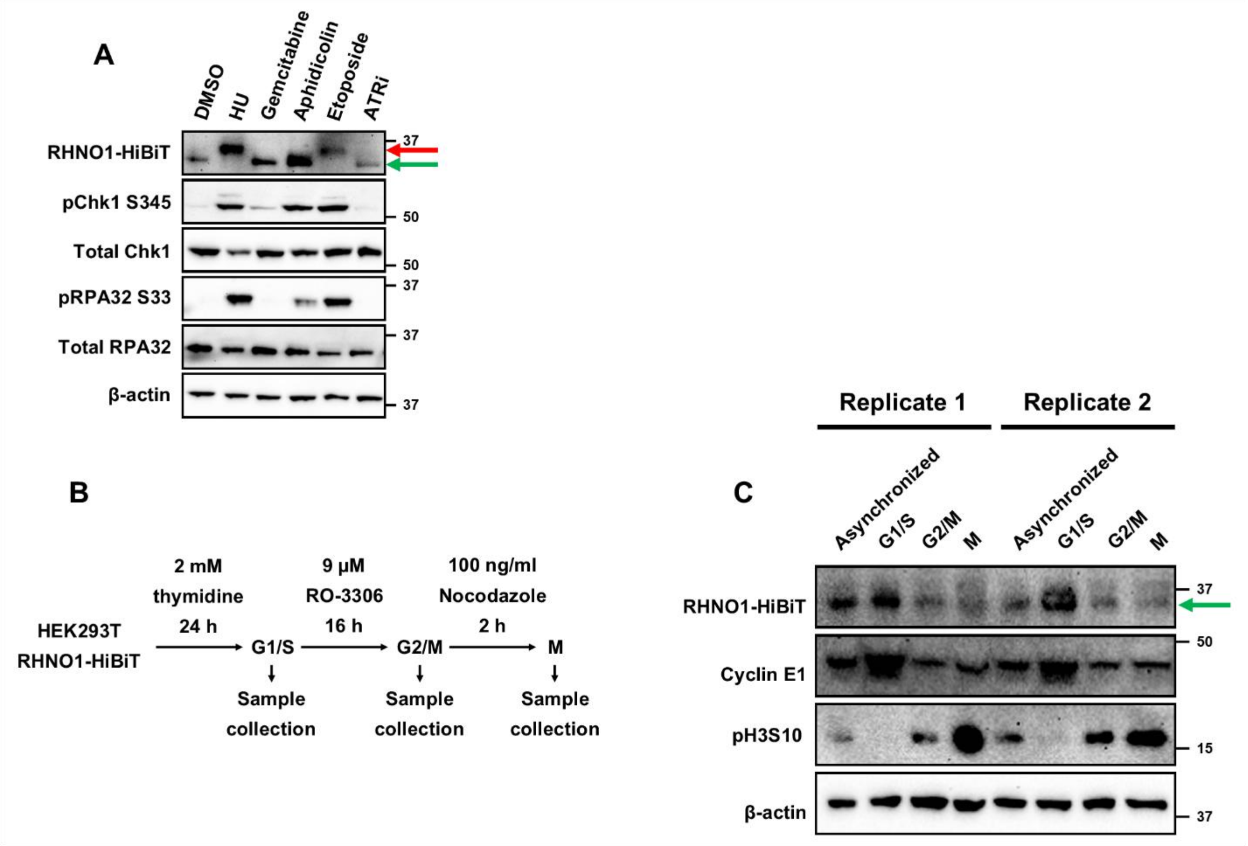

Supplementary figure 5 RHNO1 is phosphorylated in response to DNA replication stress inducers and mainly expressed during G1/S phase of cell cycle. **A** Western blotting of RHNO1-HiBiT protein in response to a panel of DNA damaging agents for 24 hours. **B** Schematic of cell cycle synchronization experiment using thymidine, RO-3306, and nocodazole. **C** Western blot analysis of RHNO1-HiBiT protein in different cell cycle phases.

bioRxiv preprint doi: https://doi.org/10.64898/2026.05.22.727300; this version posted May 24, 2026. The copyright holder for this preprint (which was not certified by peer review) is the author/funder, who has granted bioRxiv a license to display the preprint in perpetuity. It is made available under a CC-BY 4.0 International license.

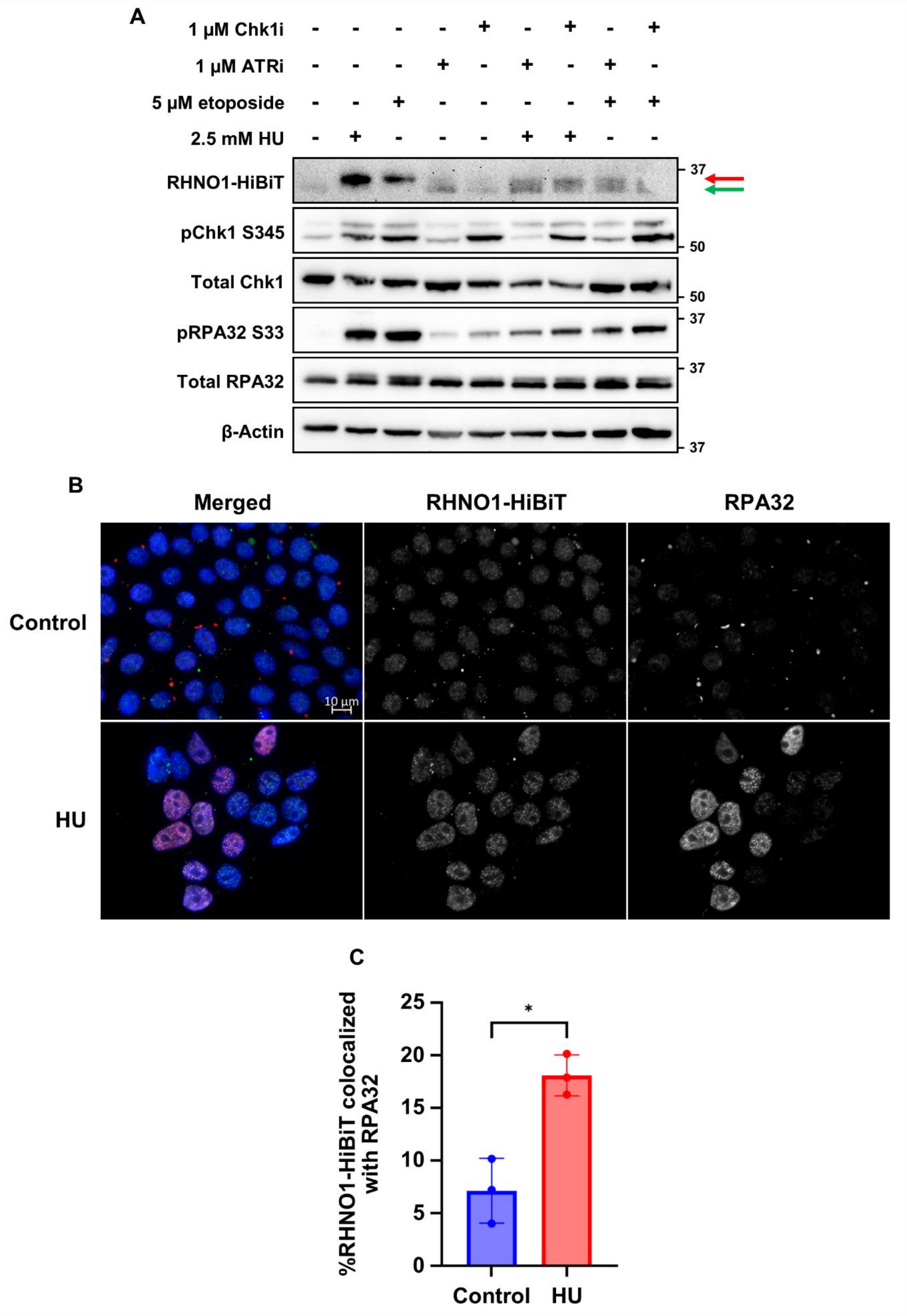

716

bioRxiv preprint doi: https://doi.org/10.64898/2026.05.22.727300; this version posted May 24, 2026. The copyright holder for this preprint (which was not certified by peer review) is the author/funder, who has granted bioRxiv a license to display the preprint in perpetuity. It is made available under a CC-BY 4.0 International license.

Supplementary figure 6 Phosphorylation of RHNO1 under replication stress condition is dependent on ATR/Chk1 kinase activities and RHNO1 form foci and colocalizes at stressed replication forks during stress condition. **A** Western blot analysis of RHNO1-HiBiT in HEK293T RHNO1-HiBiT cells treated with 2.5 mM HU or 5 μM etoposide with or without ATR or Chk1 inhibitors. Phosphorylated and unphosphorylated RHNO1 are noted by red and green arrows, respectively. **B** Representative immunofluorescence images of RHNO1-HiBiT and RPA32 foci in HEK293T RHNO1-HiBiT treated with 2.5 mM HU for 24 h. **C** Colocalization analysis of RHNO1-HiBiT and RPA32 foci in HEK293T RHNO1-HiBiT cell line. Data is presented as mean ± SD of at least three independent experiments. Statistical significance was determined using unpaired Student's t test two-tailed (* $p < 0.05$, ** $p < 0.01$). The scale bar represents 10 μm.

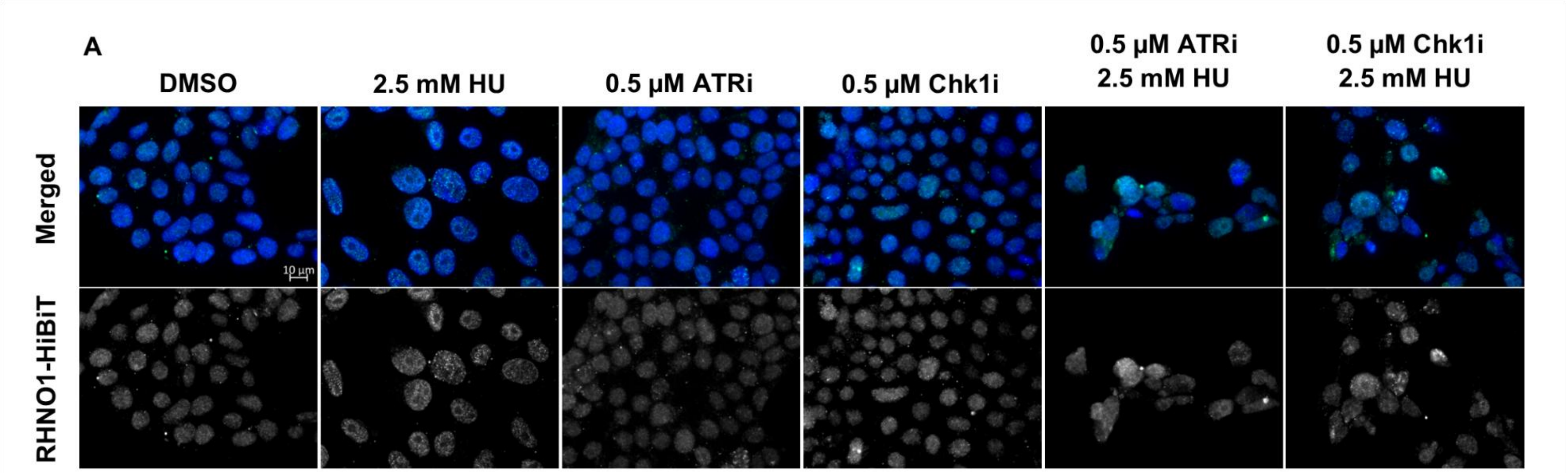

Supplementary figure 7 Inhibition of ATR/Chk1 disrupts RHNO1 foci formation under replication stress. **A** Representative immunofluorescence images of HEK293T RHNO1-HiBiT treated with HU alone or in combination with ATR or Chk1 inhibitors. The scale bar represents 10 μm.

bioRxiv preprint doi: https://doi.org/10.64898/2026.05.22.727300; this version posted May 24, 2026. The copyright holder for this preprint (which was not certified by peer review) is the author/funder, who has granted bioRxiv a license to display the preprint in perpetuity. It is made available under a CC-BY 4.0 International license.

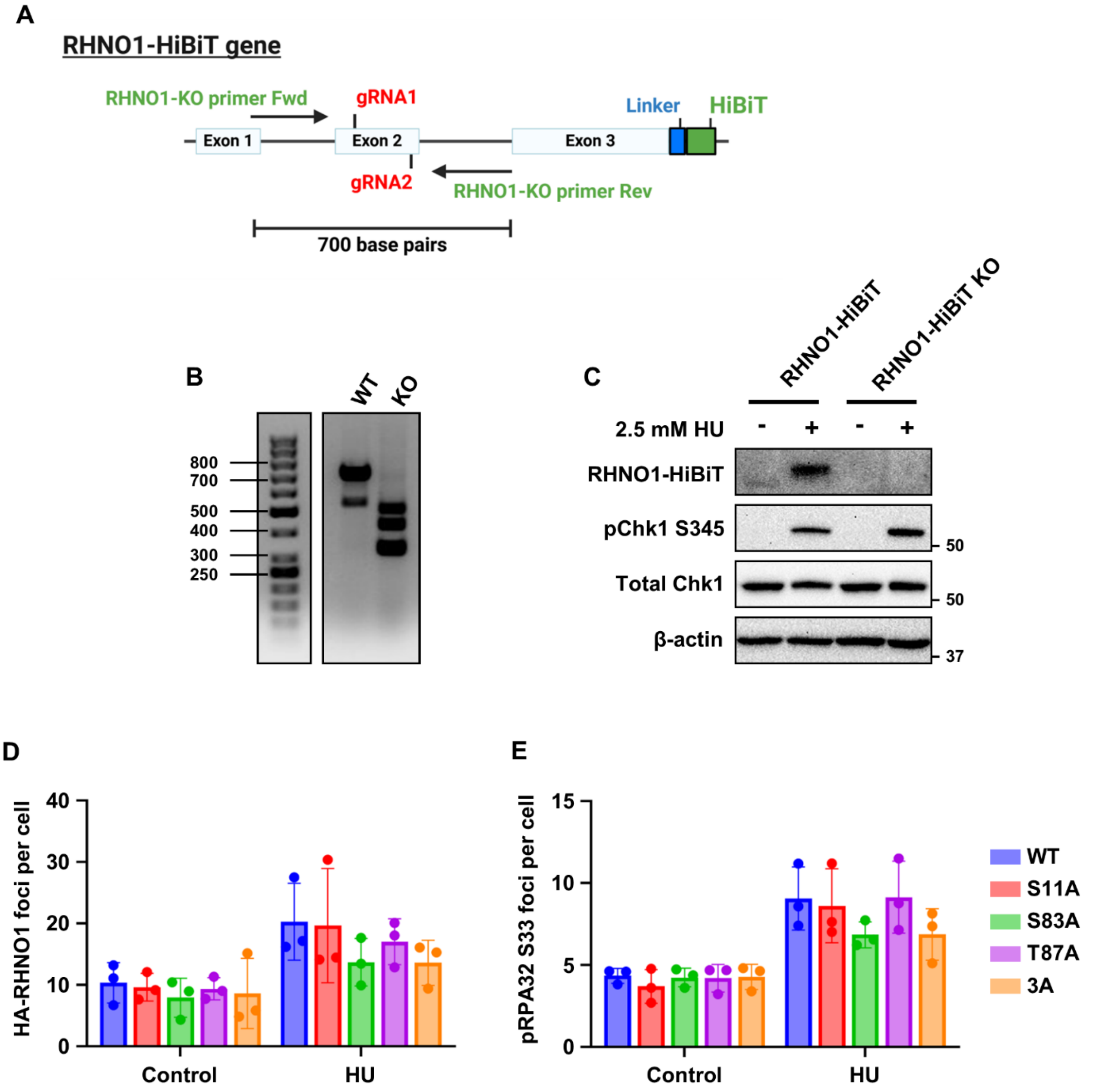

Supplementary figure 8 HA-RHNO1 re-expression in HEK293T RHNO1-HiBiT knock out cells. **A** Schematic of CRISPR-Cas9 RHNO1 knock out in HEK293T RHNO1-HiBiT cells. **B** Genomic DNA PCR analysis of parental and knock out cells. **C** Western blotting of RHNO1-HiBiT protein in parental and knock out cells. **D** and **E** quantification of HA-RHNO1 (**D**) and pRPA32 S33 foci (**E**) after 24 hours treatment of 2.5 mM HU. Data is presented as mean ± SD of at least three independent experiments.
